# Alpha-Synuclein species in oral mucosa as potential biomarkers for multiple system atrophy

**DOI:** 10.3389/fnagi.2022.1010064

**Published:** 2022-10-11

**Authors:** Yuanchu Zheng, Huihui Cai, Jiajia Zhao, Zhenwei Yu, Tao Feng

**Affiliations:** ^1^Department of Neurology, Center for Movement Disorders, Beijing Tiantan Hospital, Capital Medical University, Beijing, China; ^2^Department of Pathophysiology, Beijing Neurosurgical Institute, Beijing, China; ^3^China National Clinical Research Center for Neurological Diseases, Beijing, China

**Keywords:** multiple system atrophy, alpha-synuclein, oral mucosal cells, immunofluorescence, electrochemiluminescence

## Abstract

**Background:**

The definitive diagnosis of Multiple system atrophy (MSA) requires the evidence of abnormal deposition of α-Synuclein (α-Syn) through brain pathology which is unable to achieve *in vivo*. Deposition of α-Syn is not limited to the central nervous system (CNS), but also extended to peripheral tissues. Detection of pathological α-Syn deposition in extracerebral tissues also contributes to the diagnosis of MSA. We recently reported the increased expressions of α-Syn, phosphorylated α-Synuclein at Ser129 (pS129), and α-Syn aggregates in oral mucosal cells of Parkinson’s disease (PD), which serve as potential biomarkers for PD. To date, little is known about the α-Syn expression pattern in oral mucosa of MSA which is also a synucleinopathy. Here, we intend to investigate whether abnormal α-Syn deposition occurs in oral mucosal cells of MSA, and to determine whether α-Syn, pS129, and α-Syn aggregates in oral mucosa are potential biomarkers for MSA.

**Methods:**

The oral mucosal cells were collected by using cytobrush from 42 MSA patients (23 MSA-P and 19 MSA-C) and 47 age-matched healthy controls (HCs). Immunofluorescence analysis was used to investigate the presence of α-Syn, pS129, and α-Syn aggregates in the oral mucosal cells. Then, the concentrations of α-Syn species in oral mucosa samples were measured using electrochemiluminescence assays.

**Results:**

Immunofluorescence images indicated elevated α-Syn, pS129, and α-Syn aggregates levels in oral mucosal cells of MSA than HCs. The concentrations of three α-Syn species were significantly higher in oral mucosal cells of MSA than HCs (α-Syn, *p* < 0.001; pS129, *p* = 0.042; α-Syn aggregates, *p* < 0.0001). In MSA patients, the oral mucosa α-Syn levels negatively correlated with disease duration (*r* = −0.398, *p* = 0.009). The area under curve (AUC) of receiver operating characteristic (ROC) analysis using an integrative model including age, gender, α-Syn, pS129, and α-Syn aggregates for MSA diagnosis was 0.825, with 73.8% sensitivity and 78.7% specificity.

**Conclusion:**

The α-Syn levels in oral mucosal cells elevated in patients with MSA, which may be promising biomarkers for MSA.

## Introduction

Multiple system atrophy (MSA) is a rare and rapidly progressive neurodegenerative disease, that clinically presents with variable combination of autonomic failure, levodopa-unresponsive parkinsonism, and cerebellar ataxia (Fanciulli and Wenning, 2015). MSA can be clinically divided into two subtypes: MSA with predominant parkinsonism (MSA-P) and predominant cerebellar ataxia (MSA-C) according to their predominant clinical manifestations (Gilman et al., 2008). Currently, the diagnosis of MSA is mainly based on clinical symptoms, supplemented by magnetic resonance imaging (MRI) evidence. MRI markers of MSA are as follows: atrophy of putamen, middle cerebellar peduncle, pons, and cerebellum; “hot cross bun” sign; increased diffusivity of putamen and middle cerebellar peduncle. Due to overlapping characteristics with other synucleinopathies such as Parkinson’s disease (PD) and dementia with Lewy bodies (DLB), the misdiagnosis rate of MSA is high, particularly in the early stages (Joutsa et al., 2014; Cong et al., 2021). In the brain of MSA patients, the α-Synuclein (α-Syn) accumulates in the cytoplasm of oligodendrocytes and forms insoluble inclusion bodies, namely glial cytoplasmic inclusions (GCIs; Marmion et al., 2021). The definitive diagnosis of MSA requires the evidence of abnormal deposition of α-Syn in the brain through autopsy. α-Syn abnormal deposition in MSA patients is not limited to the central nervous system (CNS), but also extended to peripheral tissues, biofluids, and cells. Detection of α-Syn abnormal deposition in peripheral tissues may be a promising biomarker for MSA.

Studies reported α-Syn abnormal deposition in blood, olfactory mucosa, saliva, salivary glands, skin, colon, and sural nerves of MSA patients. Liu et al. found the increased α-Syn and α-Syn aggregates levels in erythrocyte membranes of MSA patient than those in healthy controls (HCs; Liu et al., 2019). Recently, Luan et al. used alpha-synuclein real-time quaking-induced conversion (α-syn RT-QuIC) assay to detect the seeding activity of pathological α-syn in the saliva (Luan et al., 2022). 11 of 18 patients with clinical probable MSA displayed positive seeding activity, whereas 2 of 36 controls showed positive seeding activity. MSA patients showed higher seeding activity of pathological α-syn in the saliva than controls. Some groups also found the positive seeding activity of α-Syn in olfactory mucosa of MSA patients (De Luca et al., 2019; Bargar et al., 2021). Skin and colon biopsies revealed the abnormal deposition of phosphorylated α-Syn in MSA patients (Pouclet et al., 2012; Donadio et al., 2018). However, most of the sampling methods are either invasive or of poor compliance. A non-invasive sampling assay with high diagnostic efficiency is in urgent need for the diagnosis of MSA. We recently reported the increased expressions of α-Syn, phosphorylated α-Synuclein at Ser129 (pS129), and α-Syn aggregates in oral mucosal cells of PD, which serve as potential non-invasive biomarkers (Zheng et al., 2022). These results suggested that oral mucosal cells may be the ideal source of biomarker for synucleinopathies including MSA. To date, little is known about the α-Syn expression pattern in oral mucosa of MSA. Whether there are differential α-Syn species expressions in oral mucosal cells between MSA patients and HCs deserves further study.

In the current study, we intend to explore the expressions of α-Syn, pS129, and α-Syn aggregates in oral mucosal cells from MSA patients and age-matched HCs, and to determine the diagnostic performance of α-Syn species in oral mucosa for MSA. We also aim to determine the relationship between oral mucosal α-Syn levels and disease severity.

## Materials and methods

### Study design and subjects

Patients with MSA and age-matched HCs were recruited from Beijing Tiantan Hospital, Capital Medical University between November 2021 and May 2022. All patients were diagnosed by a movement disorders specialist (T. F) and fulfilled the MSA criteria (Gilman et al., 2008). The exclusion criteria were as follows: (1) secondary parkinsonism due to cerebrovascular, hypoxia, trauma, infection, metabolic or systemic disease affecting the CNS; (2) other Parkinson-plus syndromes, such as DLB, PSP, and corticobasal degeneration (CBD); (3) severe systemic disorders and oral mucosa diseases. According to the dominant clinical manifestations, MSA patients were divided into MSA-P and MSA-C subtypes. HCs were excluded if they had a diagnosis of the neurological disease, a family history of movement disorders, severe psychiatric disorders, severe systemic disorders, or oral mucosa diseases.

We collected clinical and demographic data including age, gender, disease duration, predominant motor symptoms, MMSE, MoCA and orthostatic hypotension (OH). Neurogenic orthostatic hypotension is defined as a 30 mmHg systolic blood pressure (BP) decrease usually accompanied by a 15 mmHg diastolic BP drop and a heart rate (HR)/SBP ratio 0.5 bpm/mmHg within 3 min of standing or head-up tilt (HUT; Gilman et al., 2008). For MSA patients, disease duration and MDS-UPDRS III (Carmona-Abellan et al., 2021) were used to assess the disease severity. The study was approved by the Ethics Committee of Beijing Tiantan Hospital, Capital Medical University. All participants provided written informed consent.

### Oral mucosa sampling and preparation

Participants rinsed their mouths with sterile saline before sampling to exclude the effects of oral food debris and saliva contamination. Immediately after rinsing, small-headed cytobrush (2 cm head length; Thomas et al., 2009) was used to collect oral mucosa samples from bilateral inner buccal mucosa, respectively. The right-sided oral mucosa samples were used for electrochemiluminescence immunoassays, and the left-sided oral mucosa samples were used for immunofluorescence stains.

The collection and procession of oral mucosal cell samples for electrochemiluminescence (ECL) assay: the operator holds the cytobrush and makes 30 circular motions of the brush head, then immerses the brush head in a 1.5 ml tube filled with 200 μl RIPA buffer (Applygen, cat. no. C1053 +). The cytobrush head was removed after vortexing for 1 min. Then, the oral mucosal cell suspension was sonicated for 1 min and centrifuged at 12,000*g* and 4°C for 10 min. Transfer the supernatant to a new tube and discard the pellet at the bottom. Samples were stored at −80°C. The bicinchoninic acid (BCA) protein assay kit (Pierce/Thermo Fisher Scientific, Rockford, IL, United States) was used to assess the total protein levels of oral mucosal cell samples.

The collection and procession of oral mucosal cell samples for immunofluorescence stains: the operator holds the cytobrush and makes 5 circular motions of the brush head, then immerses the brush head in a 1.5 ml tube with 1 ml Phosphate-buffered saline (PBS). The cytobrush was removed after vortexing tubes for 1 min. The tube was centrifuged at 2,000*g* and 4°C for 20 min. Remove the supernatant, then resuspend the cell mass with 1.5 ml PBS. Add 70 μl cell resuspension to each funnel, mount the slide and funnel, then cytocentrifuge cell suspension onto microscope slides by cytospin (CYTOSPIN IV, AHSI, Italy) at 700 rpm for 6 min. The slides were then stored at −20°C.

### Oral mucosal cell immunofluorescence staining

#### Immunofluorescence staining with α-Syn species antibodies

The slide was fixed in 4% paraformaldehyde for 10 min and then washed with PBS. After rinsing, the slide was permeabilized in 1% Triton X-100 for 10 min and then incubated in 5% Bovine serum albumin blocking solution (Sigma, Poole, United Kingdom). We evaluated the expression of α-Syn species in oral mucosa samples using MJFR1 (ab138501, Abcam, Cambridge, MA, United States), which is specific for full-length human α-Syn protein. PS129 antibody (cat825701, BioLegend, San Diego, CA, United States) is a synthetic peptide-specific antibody, which corresponds to the α-Syn phosphorylated at serine 129. Using MJFR-14-6-4-2(ab209538, Abcam, Cambridge, MA, United States), the expression of α-Syn aggregates was determined. Primary antibodies were diluted 1:500 in blocking solution and incubated overnight at 4°C. After incubation, the slide was washed with PBST buffer (PBS with 0.05% Tween^®^ 20). The sections were then incubated with Alexa-conjugated secondary antibodies for α-Syn, pS129, and α-Syn aggregates, respectively: donkey anti-rabbit Alexa Fluor 488 (diluted 1:500, ab150077, Abcam, Cambridge, MA, United States), donkey anti-mouse Alexa Fluor 647 (diluted 1:500, ab150107, Abcam, Cambridge, MA, United States) and donkey anti-rabbit Alexa Fluor 647 (diluted 1:500, ab150075, Abcam, Cambridge, MA, United States) for 1 h at room temperature. Sealed with a DAPI-containing anti-fluorescence quencher (S2110 Solarbio, Beijing, CHINA) and imaged with Zeiss LSM 700 confocal microscope (Carl Zeiss Microscopy GmbH, Oberkochen, Germany) using 40 × objective.

#### Double immunofluorescence staining with ThT and MJFR-14-6-4-2

The slides were fixed in 4% paraformaldehyde for 10 min and then washed with PBS for three times. After rinsing, slides were permeabilized in 1% Triton X-100 for 15 min. After three washings of 5 min each, slides were incubated in 5% Bovine serum albumin blocking solution (Sigma, Poole, United Kingdom) for 2 h. After blocking, the slides were incubated overnight with 0.05% Thioflavin T (ThT) and MJFR-14-6-4-2 (diluted 1:500, ab209538, Abcam, Cambridge, MA, United States). After overnight incubation, the slides were incubated with donkey anti-rabbit Alexa Fluor 647 (diluted 1:500, ab150075, Abcam, Cambridge, MA, United States) for 1 h at room temperature. Nuclei were stained with DAPI (0.2 μg/ml) for 5 min. Slides observation was performed with a Zeiss LSM 880 confocal microscope (Carl Zeiss Microscopy GmbH, Oberkochen, Germany) using 40 × objective.

### Electrochemiluminescence immunoassays

The ECL assay of oral mucosal cells was made as described previously (Zheng et al., 2022), with minor modifications. The Meso Scale Discovery (MSD, Rockville, MD, United States) U-Plex plates were used for the quantification of oral mucosa-derived α-Syn, pS129, and α-Syn aggregates. Recombinant unphosphorylated a-Syn monomers (RP-001, Proteos, Inc., Kalamazoo, MI, United States), phosphorylated α-Syn monomers (RP-004, Proteos, Inc.) and filaments (RP-002, Proteos, Inc.) were used as the standard proteins for three a-Syn species assay, respectively. The NanoDrop OneC spectrophotometer (Thermo Scientific, Waltham, MA, United States) is used to measure the standard protein concentration. Standard protein was then diluted between 1,000 pg./ml and 1.37 pg./ml in 3-fold serial dilutions with Diluent 35 (D35, MSD, Rockville, MD, United States). Anti-α-Syn clone 42 (624096, BD Bioscience, San Jose, CA, United States) was labeled with Sulfo-TAG and used as detection antibodies for all a-Syn species. Recombinant anti-α-Syn MJFR-1 (ab 138,501, Abcam, Cambridge, MA, United States), recombinant anti-α-Syn aggregate MJFR-14 (ab209538, Abcam, Cambridge, MA, United States), and anti-phosphorylated α-Synuclein at Ser129 (825,701, BioLegend, San Diego, CA, United States) antibodies were biotinylated and used as capture antibodies. Capture antibodies were coated on plates and incubated for 1 h with 600 rpm shaking at room temperature. After rinsing three times with 150 μl wash buffer (MSD, Rockville, MD, United States), plates were blocked with 150 μl D35 for 1 h with 600 rpm shaking at room temperature and then rinsed. The oral mucosal cell protein-containing samples were diluted in D35 (protein-containing samples: D35, 25 μl:30 μl). Diluted samples together with standard proteins were loaded 50 μl/well and incubated for 1 h while shaking at 600 rpm. For α-Syn, pS129 detection, overnight incubation at 4°C is needed. After incubation, the detection antibody solution (1 μg/ml) was loaded and incubated for 1 h with 600 rpm shaking and then rinsing with wash buffer for three times. Immediately after rinsing, 150 μl 2 × Reading Buffer (MSD, Rockville, MD, United States) was loaded and the plates were analyzed in a Sector Imager 6000 (MSD, Rockville, MD, United States).

### Statistical analysis

The statistical analysis was conducted with SPSS 26.0 (IBM, Chicago, IL, United States) and GraphPad Prism 8 software (GraphPad Software, La Jolla California United States). Prior to analysis, α-Syn, pS129, and α-Syn aggregates concentrations were normalized to total oral mucosal cell protein levels. Non-parametric Mann–Whitney *U* test was performed to compare group difference. The chi-square test was used to compare the gender ratio between the two groups. Spearman’s rank correlation coefficient was employed to test the correlation between α-Syn species levels and disease severity. *p* < 0.05 was considered significant. Binary logistic regression was used to create a multivariable logistic regression model suited for MSA diagnosis. We evaluated the area under the ROC curve and derived the Youden index maxima (sensitivity + specificity −1) to determine the optimal cutoff value for MSA patients and HCs.

## Results

### Demographic and clinical features

A total of 89 subjects were included in this study. The cohort consisted of 42 MSA patients (23 patients with MSA-P and 19 patients with MSA-C) and 47 age-matched HCs. The demographic and clinical features of all subjects were listed in Table 1. There were no statistically significant differences in gender distribution and age between total MSA and HCs. While there were significant differences in Mini-mental State Examination (MMSE) and Montreal Cognitive Assessment (MoCA) scores between MSA and HCs (*p*_MMSE_ < 0.001, *p*_MoCA_ < 0.001). The median disease duration of MSA patients was 2 years (range 4 months–7.5 years) and median Movement Disorder Society Unified Parkinson’s Disease Rating Scale Part III (MDS-UPDRS III) score was 40.5 (range 21–83). For the MSA subtypes, the age and MDS-UPDRS III scores were higher in MSA-P patients compared with MSA-C (*p*_age_ = 0.023, *p*_MDS-UPDRS III_ = 0.020). There were no significant differences in gender distribution, disease duration, MoCA, MMSE scores, and orthostatic hypotension between MSA-C and MSA-P.

**Table 1:** Demographic and clinical features.

Group	MSA, *n* = 42	MSA-C, *n* = 19	MSA-P, *n* = 23	HCs, *n* = 47	*p*	
					MSA vs HCs	MSA-C vs MSA-P
Females/Males	21/21	8/11	13 /10	33/14	0.051	0.352
Age (year)	62 [54.75, 69]	56 [52, 67]	65 [57, 70]	57 [53, 66]	0.082	0.023^*^
Duration (year)	2 [1, 3.125]	1.5 [1, 2]	2.5 [2, 4]	NA	NA	0.067
MDS-UPDRS III	40.5 [31, 48.5]	36 [29, 43]	43 [36, 57]	NA	NA	0.020^*^
MoCA	21 [19, 25]	22 [19, 26]	21 [19, 24]	25 [24, 27]	<0.001^*^	0.395
MMSE	26 [24, 28]	26 [25, 27]	26 [23,28]	28 [28, 30]	<0.001^*^	0.721
OH/NOH	16/26	9/10	7/16	NA	NA	0.266

MSA, Multiple System Atrophy; HCs, Healthy Controls; MSA-P, Multiple System Atrophy with predominant parkinsonism; MSA-C, Multiple System Atrophy with predominant cerebellar ataxia; MMSE, Mini-mental State Examination; MoCA, Montreal Cognitive Assessment; MDS-UPDRS III, Movement Disorder Society Unified Parkinson’s Disease Rating Scale Part III; OH, Orthostatic Hypotension; NOH, No Orthostatic Hypotension; NA, not applicable. Data is represented as median [25%–75%].

* This value of *p* indicates a statistically significant difference.

### Immunofluorescence stains

**Figure 1:**
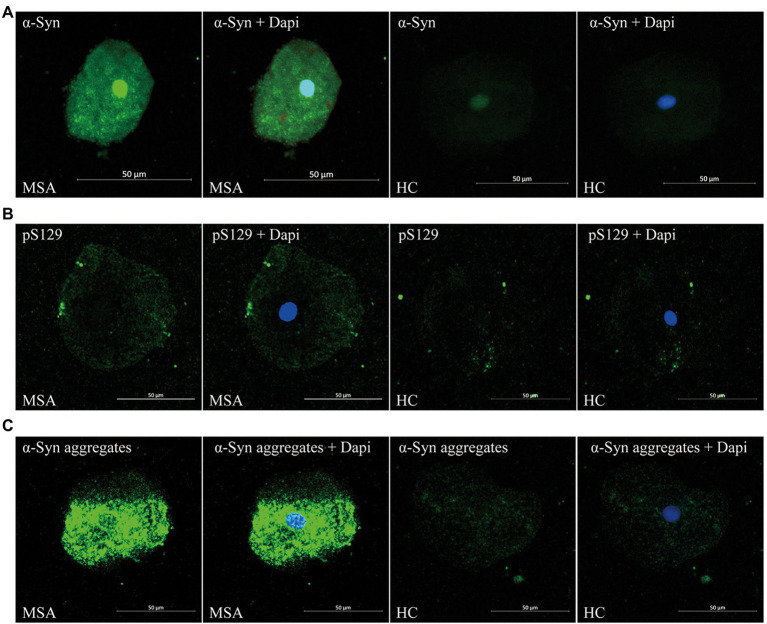
Confocal microscopy (×40) study of α-Syn **(A)**, pS129 **(B)**, and α-Syn aggregates **(C)** expression pattern in oral mucosal cells of MSA patients and controls. DAPI: bule. α-Syn: green. Scale bar: 50 μm. MSA, Multiple System Atrophy; α-Syn, α-synuclein; pS129, phosphorylated α-Syn at Ser129.

We evaluated the expression of α-Syn in oral mucosa using MJFR1, which is specific for recombinant full-length human α-Syn protein. PS129 antibody is a synthetic peptide directed toward phosphorylated serine 129 of α-Syn, which corresponds to amino acids 124–134. Using MJFR-14-6-4-2, the expression of α-Syn aggregates was determined. In oral mucosal cells of both MSA patients and HCs, α-Syn (Figure 1A), pS129 (Figure 1B), and α-Syn aggregates (Figure 1C) immunoreactive signals were detectable through confocal microscopy. Immunofluorescence imaging revealed that immunoreactive signals of α-Syn and α-Syn aggregates significantly increased in the oral mucosal cells of MSA patients compared to those of HCs, while ps129 showed a slightly higher intensity in the oral mucosal cells of MSA patients. The morphological pattern of a-syn species varies in oral mucosal cells. A-Syn aggregates showed a large granular pattern, whereas α-Syn and pS129 showed diffused or small dotted patterns.

There was no significant difference between MSA patients and HCs in the immunoreactive signal pattern or the intracellular distribution of α-Syn species. More specifically, in oral mucosal cells of both MSA patients and HCs, α-Syn showed a diffuse distribution in the nucleus and cytoplasm of oral mucosal cells. PS129 showed a dotted positivity mainly located in the cytoplasm of oral mucosal cells. Α-Syn aggregates showed a predominantly granular positivity in the nucleus and perinuclear cytoplasm of oral mucosal cells.

**Figure 2:**
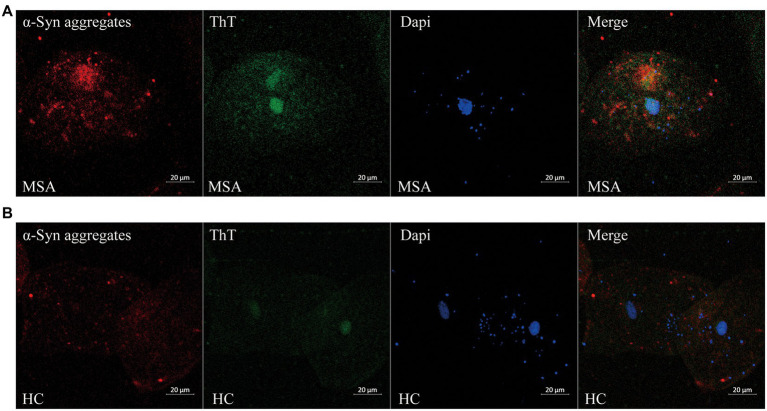
Confocal microscopy (×40) study of double immunofluorescence staining with ThT and MJFR-14-6-4-2 antibody in oral mucosal cells of MSA patients **(A)** and controls **(B)**. DAPI: bule. α-Syn aggregates: red. ThT: green. Scale bar: 20 μm. MSA, Multiple System Atrophy; α-Syn, α-synuclein; ThT, Thioflavin T.

Through confocal microscopy analysis, ThT immunoreactive signals were visible in oral mucosa cells of both MSA (Figure 2A) and control groups (Figure 2B). ThT immunoreactive signals were slightly higher in MSA group than the control group. Double immunofluorescence staining with ThT and MJFR-14-6-4-2 antibody revealed that MJFR-14-6-4-2 immunoreactive signals mainly colocalized with ThT immunoreactive signals in the cytoplasm of oral mucosal cells in MSA group (Figure 2A).

### Electrochemiluminescence assay

#### The concentrations of α-Syn species in oral mucosal cells between MSA and HCs

**Figure 3:**
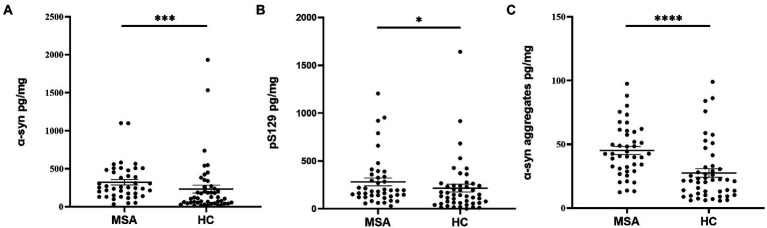
The concentrations of α-Syn, pS129, and α-Syn aggregates in oral mucosa samples of MSA patients and controls. **(A)** α-Syn, normalized to total oral mucosal cell proteins (pg/mg); ^***^p < 0.001 (Mann–Whitney *U* test); **(B)** pS129, normalized to total oral mucosal cell proteins (pg/mg); ^*^p = 0.042 (Mann–Whitney *U* test); **(C)** α-Syn aggregates, normalized to total oral mucosal cell proteins (pg/mg); ^****^p < 0.0001 (Mann–Whitney *U* test).

Before analysis, the concentrations of α-Syn, pS129, and α-Syn aggregates were all standardized to the total protein levels of oral mucosa samples. Compared with HCs, the concentration of α-Syn in oral mucosal cells was significantly higher of MSA patients (*p* < 0.001, Figure 3A; Table 2). The pS129 and α-Syn aggregates levels also increased in MSA patients compared with those in HCs (*p* = 0.042, *p* < 0.0001, Figures 3B,C; Table 2).

**Table 2:** The concentrations of α-Syn in oral mucosal cells of MSA and HCs.

Group	MSA	MSA-C	MSA-P	MSA-OH	MSA-NOH	HCs	*p*		
							MSA vs HCs	MSA-C vs MSA-P	MSA-OH vs MSA-NOH
α-Syn	253.69 [147.47, 481.28]	304.65 [176.13, 507.72]	239.11 [130.97, 454.33]	288.31 [182.30, 500.93]	250.06 [130.64, 462.04]	112.39 [47.40, 261.64]	<0.001^*^	0.294	0.422
pS129	188.92 [129.57, 342.38]	203.13 [131.96, 412.13]	163.59 [122.17, 270.60]	188.55 [124.77, 343.46]	188.92 [135.78, 350.01]	142.96 [61.88, 245.39]	0.042^*^	0.185	0.756
Α-Syn aggregates	42.53 [30.40, 60.04]	42.63 [30.90, 61.43]	40.32 [28.73, 59.8]	42.61 [29.40, 58.29]	41.60 [30.82, 60.22]	21.53 [10.29, 31.97]	<0.0001^*^	0.426	0.856

MSA; Multiple System Atrophy; HCs; Healthy Controls; MSA-P, Multiple System Atrophy with predominant parkinsonism; MSA-C, Multiple System Atrophy with predominant cerebellar ataxia; MSA-OH, MSA with orthostatic hypotension; MSA-NOH, MSA without orthostatic hypotension; α-Syn, α-synuclein; pS129, Phosphorylated α-Syn at Ser129; Data is represented as median [25%–75%].

* This value of *p* indicates a statistically significant difference.

#### The concentrations of α-Syn species in oral mucosal cells between MSA subgroups

The MSA group was further divided into MSA-C/MSA-P and MSA-OH/MSA-NOH subgroups according to clinical manifestations. There was no significant difference in α-Syn, pS129, or α-Syn aggregates levels between MSA-C and MSA-P patients (*p*_α-Syn_ = 0.294, *p*_pS129_ = 0.185, *p*_α-Syn aggregates_ = 0.426). There was neither a significant difference of α-Syn, pS129, nor α-Syn aggregates levels between MSA patients with or without orthostatic hypotension (*p*_α-Syn_ = 0.422, *p*_pS129_ = 0.756, *p*_α-Syn aggregates_ = 0.856; Table 2).

#### The correlations of α-Syn species concentrations with disease severity in MSA

**Figure 4:**
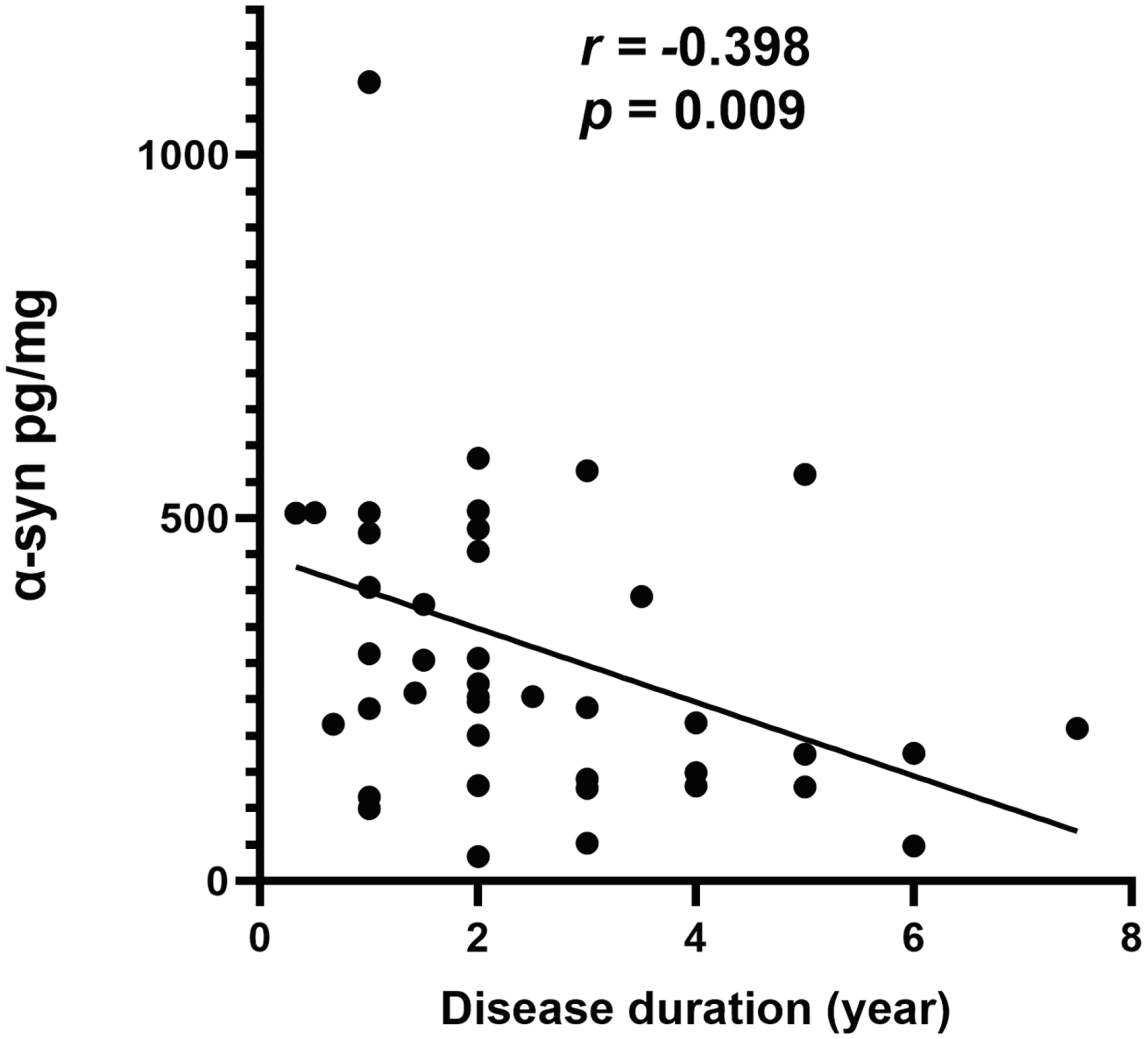
Correlation of α-syn in oral mucosa with disease duration in patients with MSA.

In MSA group, there was a significantly negative correlation between α-syn levels in oral mucosal cells and disease duration (*r* = −0.398, *p* = 0.009; Figure 4). However, α-Syn and pS129 levels in oral mucosal cells did not significantly correlate with disease duration in MSA group. The α-Syn species levels did not correlate with MDS-UPDRS III scores or cognitive scores in patients with MSA.

#### ROC curve analysis of α-Syn species in oral mucosal cells between MSA and HCs

**Figure 5:**
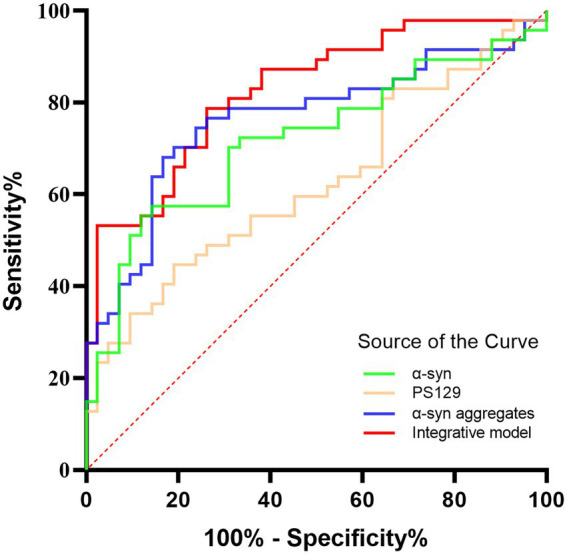
The ROC curves for α-Syn, pS129, α-Syn aggregates, and the integrative model. Green curve: α-Syn (AUC = 0.718); Yellow curve: pS129 (AUC = 0.625); Blue curve: α-Syn aggregates (AUC = 0.763); Red curve: the integrative model including α-Syn, pS129, and α-Syn aggregates (AUC = 0.825).

Receiver operating characteristic (ROC) analysis was performed to assess the diagnostic performance of α-Syn species in oral mucosal cells for MSA. α-syn levels discriminated MSA from HCs with a sensitivity of 88.1% and a specificity of 55.3%, and the area under curve (AUC) was 0.718. PS129 levels discriminated MSA from HCs with a sensitivity of 81.0% and a specificity of 44.7% (AUC = 0.625). Α-Syn aggregates discriminated MSA from HCs with a sensitivity of 69.0% and a specificity of 76.6% (AUC = 0.763). The binary logistic regression with forward like ratio was used to create a multivariable logistic regression model based on age, gender, and levels of α-Syn species in oral mucosal cells. The AUC of this integrative model was 0.825, with 73.8% sensitivity and 78.7% specificity (Figure 5).

## Discussion

The current study demonstrated the differential expression of α-Syn, pS129, and α-Syn aggregates in oral mucosal cells between MSA patients and HCs. The main results are as follows: (1) immunofluorescence images showed detectable α-Syn species immunoreactive signals in oral mucosal cells of both MSA patients and HCs, with higher immunoreactive signals of α-Syn, pS129, and α-Syn aggregates in oral mucosal cells of MSA patients; (2) ECL assays demonstrated that the α-Syn, pS129, and α-Syn aggregates levels in oral mucosal cells were increased in MSA group compared with HCs, further supporting the immunofluorescence results; (3) the level of α-Syn in oral mucosal cells negatively correlated with the disease duration in MSA patients.

α-Syn is a small (14 kDa) and highly conserved acidic protein normally expressed in neurons (Rcom-H’cheo-Gauthier et al., 2016). Abnormal deposition of α-Syn plays a critical role in the pathogenesis of MSA (Clayton and George, 1999; Serratos et al., 2022). In MSA patients, α-Syn mainly accumulates in the cytoplasm of oligodendrocytes of the CNS and forms glial cell inclusion bodies. In fact, α-Syn not only deposits in the CNS of MSA, but also deposits in peripheral nervous system. Zhe Rong et al. reported the deposition of phosphorylated α-syn in the Schwann cells (SCs), a kind of peripheral glial cells, of sural nerves in MSA patients (Rong et al., 2021). Donadio et al. reported the abnormal deposition of pS129 in cutaneous nerve fibers of MSA patients, mainly in the somatosensory fibers of the subcutaneous plexus (Donadio et al., 2018). Some studies also reported the deposition of α-Syn in peripheral non-nervous tissues, biofluids, and cells of MSA (Lee et al., 2017; Ma et al., 2019). Liu et al. found that MSA patients had higher α-Syn and α-Syn aggregates levels in the erythrocyte membranes than HCs (Liu et al., 2019). A meta-analysis revealed that MSA patients had higher plasma α-Syn level than controls (Yang et al., 2018). Several studies also reported the depositions of α-Syn in the salivary glands and olfactory mucosal cells of MSA patients (De Luca et al., 2019; Cao et al., 2020; Bargar et al., 2021). These studies suggest that α-Syn not only exists in the CNS, but also exits in peripheral biofluids, tissues, and cells of MSA patients, which is consistent with the current study.

In the current study, pS129 and α-Syn aggregates immunoreactive signals were also detectable in control group, indicating that pS129 and α-Syn aggregates were expressed in normal controls’ oral mucosal cells. Several recent studies also reported the detectable pS129 immunoreactive signals in normal controls’ peripheral tissues of healthy controls, such as submucosal plexus and skin (Barrenschee et al., 2017; Wang et al., 2020). As for the α-Syn aggregates expression in controls, Mazzetti et al. recently found that PD patients showed higher α-Syn aggregates expression in skin than healthy controls. Using proximity ligation assay, skin α-Syn aggregates expression was detectable in about 2/3 of the included healthy controls (Mazzetti et al., 2020). These previous studies suggested that pathological α-Syn, pS129, and α-Syn aggregates were detectable in peripheral tissues of normal controls but with a lower level, which was consistent with the current study.

ThT is a benzothiazole dye that exhibits enhanced fluorescence upon binding to β-sheet-rich protein aggregate including Abeta fibrils, α-Syn filaments, PrP (Sc) (Khurana et al., 2005), which does not specifically demonstrate the enrich of α-Syn fibrillar aggregates. Through confocal microscopy analysis, ThT immunoreactive signals were detectable in oral mucosal cells, which indicates the potential protein aggregation in oral mucosal cells. Colocation of ThT and MJFR-14-6-4-2 immunoreactive signals indicates the presence of the α-Syn fibrils in oral mucosal cells. However, due to the minimal level of intracellular α-Syn aggregate in the mucosal cells and the lack of α-Syn oligomer or fibril-specific antibody, it is very difficult to in-depth investigate the differences between soluble oligomers and the presence of fibrils in oral mucosal cells in the current study.

The mechanism of the increased α-Syn expression in oral mucosal cells of MSA is currently unknown. In order to avoid contamination with saliva, included participants were asked to rinse their mouths with sterile saline 3 times in the current study. Immediately after rinsing, cytobrush was used to collect oral mucosa samples. Also, before the placement of cells on the slides, oral mucosa cells were washed with PBS and centrifuged at 2,000*g*. Then, the supernatant was removed, so that only mucosa cells were placed onto slides. The method of oral mucosa cell sampling used in the current study was based on the buccal micronucleus cytome assay (Thomas et al., 2009). Through confocal microscopy, the detected a-syn species were located in buccal micronucleus cells. Therefore, we believe that a-syn species detected were derived from oral mucosa cell rather than saliva gland cells or nervous fibers. Numerous researchers have focused on the alterations of oral mucosal cells in neurodegenerative disorders. As for the DNA content, DNA content of oral mucosal cells in certain neurodegenerative disorders, such as AD, is significantly higher than the control group (Francois et al., 2014). Some researchers reported that the levels of the Tau protein and the transcripts of p-Tau and Tau are increased in the oral mucosal cells of patients with cognitive impairment (Hattori et al., 2002; Arredondo et al., 2017). The above studies indicated that α-Syn may also have increased expression in oral mucosal cells in synucleinopathies. Our previous study reported the increased expressions of α-Syn species in oral mucosal cells of PD patients. In the present study, we demonstrated that abnormal α-Syn species deposition also occurred in oral mucosal cells of MSA. The meaning of abnormal deposition of α-Syn species in oral mucosal cells of synucleinopathies remains unknown, mainly because the physiological role of α-Syn in oral mucosal cell is uncertain. It is now widely accepted that abnormal α-Syn deposition in the CNS causes neurodegeneration (Burmann et al., 2020). Whether the increased level of α-Syn in oral mucosal cells of synucleinopathies is the cause or consequence of neurodegeneration requires further study.

The current study showed that the level of α-Syn in oral mucosal cells negatively correlated with the disease duration in MSA patients (*r* = *−*0.398, *p* = 0.009). However, α-Syn in oral mucosal cells did not correlate with age or MDS-UPDRS III scores. This is the first study on α-Syn expression levels in oral mucosal cells of MSA, the relationship between α-Syn levels in oral mucosal cells and disease progression remains inconclusive. A previous study revealed that α-syn oligomers/protein ratio in erythrocyte membrane negatively correlated with disease duration in MSA (*r* = −0.336; *p* = 0.009). One possible explanation is that the majority of the α-syn transferred to glial cell inclusions as MSA progresses, while lesser α-syn diffuses from the brain to the peripheral oral mucosal cells (Motter et al., 1995; Sun et al., 2014). We presume that α-Syn levels may decrease in peripheral tissues with the progression of MSA. Our findings should be confirmed by future larger and longitudinal studies.

The ECL assays used for detecting α-Syn, pS129, and α-Syn aggregates of oral mucosa were established previously (Zheng et al., 2022) and have been applied to detect α-Syn, pS129, and α-Syn aggregates in erythrocytes (Tian et al., 2019; Yu et al., 2022). Previous studies have validated the high sensitivity and reproducibility of ECL detection of disease-associated proteins on CSF samples (Kruse et al., 2017). As the signal generated is highly proportional to the large number of detection antibodies conjugated to the Sulfo-TAG marker, the ECL system has the advantage of a wide dynamic range and high sensitivity. Also, it has the advantage of shorter experiment time and the lower clinical sample volumes required. The above makes ECL an appropriate technique for detecting the trace proteins in peripheral biological specimens.

Recent developed ultrasensitive protein amplification assays, such as RT-QuIC and protein misfolding cyclic amplification (PMCA), offered an ultrasensitive approach for pathological α-syn assays (Parnetti et al., 2019; Koga et al., 2021). This assay induces conversion of normal α-syn in samples to misfolded α-syn, allowing amplification and detection of trace amounts of pathological α-syn in tissues or biofluids (Nakagaki et al., 2021). Previous studies showed that the CSF α-syn RT-QuIC had high specificity (82.3%–100%) and sensitivity (84%–100%) for the diagnosis of PD and other synucleinopathies (Nakagaki et al., 2021). Subsequent studies used RT-QuIC for the detection of pathological α-syn in various biospecimens, such as submandibular gland (sensitivity: 100%; specificity: 94%; Manne et al., 2020a), olfactory mucosa (sensitivity: 46%–69%; specificity: 90%–91%; De Luca et al., 2019; Bargar et al., 2021; Stefani et al., 2021), skin (sensitivity: 75%–100%; specificity: 83%–100%; Hong et al., 2010; Manne et al., 2020b; Donadio et al., 2021; Kuzkina et al., 2021), and saliva (sensitivity: 61.1%–76.0%; specificity: 94.4%; Luan et al., 2022). The detection of pathological α-Syn using RT-QuIC yielded high sensitivity and specificity in both synucleinopathies and the prodromal stage of synucleinopathies. The seeding activity of pathological α-syn in oral mucosa cells should be further studied by RT-QuIC or PMCA assays which might be ideal approaches to the detection of trace amounts of pathological α-syn in oral mucosal cells.

Detecting α-Syn species in the oral mucosa as the biomarkers for MSA have several advantages over other peripheral samples. First of all, compared with invasive procedures such as lumbar puncture, blood drawing, skin, salivary gland, and gastrointestinal biopsies, sampling of oral mucosal cells is a noninvasive and safe procedure. Moreover, the oral mucosal cells renew every 7–21 days, repeated sampling in a short period of time can be achieved (Thomas et al., 2009).

As a relatively easy-to-obtain biospecimen, there were numbers of studies focused on salivary α-Syn species as biomarkers for synucleinopathies. Devic et al. reported that the salivary α-Syn tends to decrease in PD patients compared with controls (Devic et al., 2011). While, Goldman et al. reported that salivary α-Syn did not differ between PD and controls (Goldman et al., 2018). Vivacqua et al. detected lower α-Syn and higher oligomeric α-Syn in saliva of PD patients than in healthy controls, the sensitivity and the specificity of salivary oligomeric α-Syn/α-Syn ratio ware 69.77% and 95.16% in distinguishing PD from healthy controls (Vivacqua et al., 2016, 2019). Cao et al. reported the higher level of α-Syn in salivary extracellular vesicles (EVs) of PD patients than that of HCs (Cao et al., 2020). Oligomeric α-Syn in salivary EVs distinguished PD from HCs with sensitivity of 92% and specificity of 86%. Recently, Luan et al. used alpha-synuclein real-time quaking-induced conversion (α-Syn RT-QuIC) assay to detect the seeding activity of pathological α-Syn in the saliva. MSA patients showed higher seeding activity of pathological α-Syn in the saliva than controls (Luan et al., 2022). Salivary α-syn RT-QuIC displayed a sensitivity of 61.1% and specificity of 94.4% in distinguishing MSA from controls. In the current study, the sensitivity and specificity of α-Syn in oral mucosal cells for MSA diagnosis were 73.8% and 78.7%, separately. Further study is needed to determine the optimal biomarker for PD: α-Syn in saliva or oral mucosal cells. In my opinion, the salivary α-Syn may not derive from oral mucosa cells. In the previous studies focused on salivary α-syn, saliva was always centrifuged after sampling and only supernatant was reserved. Oral mucosal cells as well as debris and other tissues were removed. The main contribution on salivary a-syn may be secreted α-syn from salivary glands. Cause abnormal deposition of α-syn has been reported by many groups (Andréasson and Svenningsson, 2021). The origins of salivary a-syn still need further validation. As is known, PD predominantly affects the elderly. Salivary gland atrophy in the elderly could reduce saliva production. Trihexyphenidyl, a commonly used PD drugs, can also cause side effects of reduced saliva production. Thus, oral mucosal cells may have an advantage over saliva in terms of sampling.

This study has some limitations. Above all, we did not include patients with PD or DLB in the current study because of the lack of other synucleinopathies patients. We are now accelerating our efforts to collect enough samples to explore the expression patterns of oral mucosal α-Syn among PD, MSA, and DLB patients. Furthermore, the current study was a cross-sectional study, and prospective studies are required to determine whether α-Syn in oral mucosal cells was a biomarker of disease progression for MSA.

## Conclusion

The current study demonstrated the differential expression of α-Syn, pS129, and α-Syn aggregates in oral mucosal cells between MSA patients and HCs, which may potentially serve as a diagnostic biomarker for MSA. Moreover, α-Syn level in oral mucosa is inversely correlated with disease progression. Further longitudinal cohort studies are needed to validate α-Syn in oral mucosal cells as a progression biomarker for MSA.

## Data availability statement

The raw data supporting the conclusions of this article will be made available by the authors, without undue reservation.

## Ethics statement

The studies involving human participants were reviewed and approved by the Ethics Committee of Beijing Tiantan Hospital, Capital Medical University. The patients/participants provided their written informed consent to participate in this study.

## Author contributions

TF and ZY designed the study. HC and YZ performed the measurements and data analysis and wrote the manuscript. JZ and HC contributed to sample collection and preparation. All authors contributed to the article and approved the submitted version.

## Funding

This research was funded by the National Natural Science Foundation of China (grant numbers 82071422, 81901151, and 82020108012) and Beijing Municipal Natural Science Foundation (grant number 7212031).

## Conflict of interest

The authors declare that the research was conducted in the absence of any commercial or financial relationships that could be construed as a potential conflict of interest.

## Publisher’s note

All claims expressed in this article are solely those of the authors and do not necessarily represent those of their affiliated organizations, or those of the publisher, the editors and the reviewers. Any product that may be evaluated in this article, or claim that may be made by its manufacturer, is not guaranteed or endorsed by the publisher.
